# Properties of a short questionnaire for assessing Primary Care experiences for children in a population survey

**DOI:** 10.1186/1471-2458-11-285

**Published:** 2011-05-09

**Authors:** Silvina Berra, Kátia B Rocha, Maica Rodríguez-Sanz, M Isabel Pasarín, Luis Rajmil, Carme Borrell, Barbara Starfield

**Affiliations:** 1 Catalan Agency for Health Information, Assessment and Quality (CAHIAQ), Barcelona, Spain; 2 National Research and Technology Council (CONICET), Córdoba, Argentina; 3 Agència de Salut Pública de Barcelona, Barcelona, Spain; 4 CIBER Epidemiología y Salud Pública (CIBERESP), Barcelona, Spain; 5 Johns Hopkins University, Baltimore, USA

## Abstract

**Background:**

The Primary Care Assessment Tool (PCAT) is an interesting set of tools for primary care research. A very short version could inform policy makers about consumer experiences with primary care (PC) through health surveys. This work aimed to investigate the validity and reliability of a selection of items from the child short edition (CS) of the PCAT.

**Methods:**

A 24 item questionnaire permitted the identification of a regular source of care and the assessment of the key attributes of first contact, ongoing care over time, coordination, services available and services received (comprehensiveness), and cultural competence. Structural validity, reliability, and construct validity were assessed using responses from 2,200 parents of a representative sample of the population aged 0 to 14 years in Catalonia (Spain) who participated in the 2006 Health Survey. Structural validity was analyzed using exploratory and confirmatory factor analyses and reliability was assessed using Cronbach's alpha. Construct validity was assessed using linear regression analysis between PC experience scores and a measure of overall user satisfaction with healthcare services.

**Results:**

A total of 2,095 (95.2%) parents provided useable responses on PC. After Confirmatory Factor Analysis (CFA), the best fitting model was a 5-factor model in which the original dimensions of first contact and ongoing care were collapsed into one. The CFA also showed a second order factor onto which all domains except services available loaded (root mean square error of approximation = 0.000; comparative fit index = 1.00). Cronbach's alpha values for one of the original scales (first-contact) was poor (alpha < 0.50), but improved using the modified factor structure (alpha > 0.70). Scores on the scales were correlated with satisfaction with healthcare services (p < 0.01), thereby providing some preliminary evidence of construct validity.

**Conclusions:**

This very short questionnaire obtained from the PCAT-CE yields information about five attributes of PC and a summary score. It has shown evidence of validity and reliability for judgments about experiences with primary care overall. If space on surveys is at a premium, the instrument could be useful as a measure of PC experiences.

## Background

The importance of primary care (PC) in making health care delivery more efficient and in tackling health inequalities has been discussed at a political level worldwide for at least the last three decades [1]. Several countries have reformed their health system with a particular emphasis on strengthening primary care. Public health researchers have made considerable efforts to assess the quality of primary care and the extent of improvements. Interest in a broader theoretical model of PC has also grown, and it has become clear that there is a need for research tools to measure important attributes of primary care from the user's point of view [2,3].

Theoretical models of primary care have stressed its structural and organizational aspects [4,5] and other attributes essential to primary contact for users and their families, focusing on the user perspective [6,7]. One of the most widely acknowledged definitions of the quality of PC services is "the provision of accessible and integrated services addressing a large majority of health care needs, developing a sustained relationship with patients, and practicing in the context of family and community" [8]. The Primary Care Assessment Tools (PCAT), developed in the United States (US), addresses these issues [9].

The PCAT is a set of questionnaires consisting of consumer-client surveys, facility surveys, and provider surveys. Their development provided a set of tools for PC evaluation that have become increasingly widely used in several countries [10-13]. The different versions include items designed to collect information on four core and three ancillary domains of PC. The core attributes are [14]: first contact, i.e. assessment of PC's role as the entry point to the health care system except in emergency situations; ongoing care, i.e. maintenance of person-focused care over time; comprehensiveness, which deals with PC's ability to make available and provide a wide range of services, including but not limited to preventive care, in response to prevalent health needs; and coordination, i.e. to support and integrate the care of problems addressed elsewhere, either by practitioners or medical records. PCAT also addresses the extent to which PC focuses on the health of individuals within the context of family (family orientation), is able to tackle community health problems (community orientation), and can deal with the need to establish relationships with people from different social groups (cultural competence).

Spain initiated substantial health care reforms in 1986, by legislating for universal tax-financed services, increasing the proportion of total health expenditure which is publicly financed, [15] and making the strengthening of primary care teams a central part of its strategy in primary care. The state-run health service provides free access to services, but approximately 10% of adult people and one third of the infant population pay for additional private healthcare coverage. Autonomous Communities have full autonomy in the health-care sector in Spain. In Catalonia, in the north-east of Spain, health interview surveys have been conducted periodically since 1983 (Barcelona city) and 1994 (Catalonia) to evaluate the population's health status and its determinants, and to assess the performance of healthcare services. In the 2006 edition of the Catalan Health Interview Survey,[16] the intention to assess Primary Health Care led to interest in implementing the PCAT. Since the expanded version of the PCAT for children (PCAT-CE) or the short version (PCAT-CS) could not be included due to limitations in the length of the survey, several items from the PCAT-CS were selected to provide information on primary care. Given that use of this subset of items represented a substantial modification to the original instrument, it was considered necessary to re-assess its psychometric characteristics. The research question addressed in this paper is whether the selected items provide a valid and reliable measure of parent experiences with PC.

## Methods

### Sample and setting

The 2006 Catalan Health Interview Survey was a cross-sectional study carried out in a representative sample of non-institutionalized residents of Catalonia. Sample selection used a multi-stage design. First, municipalities were selected according to number of inhabitants and, second, individuals were selected based on age and sex distribution from the population registry of the Statistical Institute of Catalonia (IDESCAT). The sample size for the population under 15 years was established at 2,200 infants and children. Main cases and five replacements were initially selected. The sample size was reached with 65% of main cases and 22% of first replacement cases; the remainders were mostly second replacement cases. Reasons for replacement were because parents refused the interview or were not found at the available address. The survey questionnaire was administered during home-based interviews with a proxy respondent, preferably the child's usual caregiver.

### The instrument to assess PC in Spain

The original child edition of the short, consumer version of the PCAT contains 44 items (PCAT-CS) distributed over 7 domains [17]. Five initial questions are used to identify the source of Primary Care. First contact accessibility and utilization (first contact domain), longitudinal interpersonal relationships (ongoing care domain), coordination of services (coordination domain), comprehensiveness of services available and received (comprehensiveness domain) are assessed through questions on how confidant the respondent is about their provision by the primary care source. Three ancillary domains (family centeredness, community orientation, and cultural competence) are also assessed.

To include the PCAT in the 2006 Catalan Health Interview Survey questionnaire for 0 to 14 year olds, it was necessary to select a subset of items due to limitations on space. Based on the PCAT-CS, the local working group together with one of the authors of the original version agreed on a very short version that was considered to best represent the thrust of the original questionnaire. This compromise led to the exclusion of the Community and Family Orientation domains, and the selection of a minimum of two items from the remaining domains based on their interest in covering a broader range of aspects of primary care performance. The final version included twenty-four items adapted to the Spanish context, since the Health System is homogeneous in all the Autonomous Communities in Spain. Two initial questions ask if children have a regular source of primary care or, if not, about the last general practitioner or pediatrician that they visited. Three questions characterize the regular source of care. The remaining 19 items cover five of the seven domains in the original questionnaire, including first contact (4 items, mostly addressing accessibility), ongoing care (3 items), coordination of services (a filter plus 2 items), comprehensiveness (4 items in the services available subdomain, and (3 items of the services received subdomain, one of which is only for adolescents), and cultural competence (2 items). Items and other text were translated into Catalan and Spanish, the main official languages spoken in Catalonia, following an internationally recommended process of cross-cultural adaptation to achieve semantic equivalence with the original version [18]. Further details of the selection of items and the cross-cultural adaptation have been published elsewhere [19]. The current analysis focuses only on the 17 items actually used to assess PC attributes across the full age-range for paediatric care. The 5 items used to identify and characterise the source of care were therefore excluded, together with the filter question before the coordination domain, and one item which only applies to adolescents.

All items covering the PC domains are answered on a 4-point Likert-type scale (1 = definitely not; 2 = probably not; 3 = probably yes; and 4 = definitely yes). Additional response options included "don't know" or "can't remember" [20]. The score for each domain is computed as the mean value for all items in that domain, and can range between 1 and 4.

The Catalan Health Interview Survey is an observational health survey conducted by the Department of Health of Catalonia as part of routine governmental statistics gathering. The survey complies with all relevant national legislation on the protection and processing of personal data. Data are not openly available but can be obtained on request from the Catalan Department of Health, and the entire questionnaire can be accessed in its website [16].

### Analysis

Demographic and health characteristics were described for the sub-sample of the Catalan Health Interview Survey used in the present analysis. As those who did not identify a regular source of care were excluded from the remainder of the analysis, they were compared with those included using chi-square to test for differences on demographic and health status variables.

Several factor analyses were carried out to evaluate the structural validity of this modified version of the PCAT. Factor analysis is a multivariate technique that allows the implementation of a statistical model that reproduces the item variance-covariance matrix and provides statistical information on model quality. In the first instance, we used exploratory factor analysis (EFA) to extract an increasing number of first order factors and account for observed variability without further restrictions on the model. Factors were extracted using diagonally weighted least squares (DWLS) on the item polychoric correlation matrix. Oblimin rotation was applied to allow for correlation between factors. We hypothesized that items would load onto domains in the same way as in the original questionnaire, thereby indicating the appropriateness of the underlying conceptual model for the Catalan population. Preliminary results from this initial factor analysis were further tested using Confirmatory Factor Analysis (CFA). In this case, certain restrictions were imposed on factor structure, and the presence of second order factors and the structural validity of a global score encompassing all domains were also tested. The unweighted least squares estimation method on the item polychoric correlation matrix was used to obtain p-values and standard errors robust for non-normal distributions. This method respects the ordered categorical nature of the items while providing robust estimates of factor loading. The model's overall goodness of fit was assessed using the root mean square error of approximation (RMSEA), and two incremental fit indices, the comparative fit index (CFI), and the Tucker Lewis-Index (TLI). A RMSEA under 0.06 was considered to indicate an excellent fit between the specified model and the data, whereas values of CFI and TLI were required to be over 0.95. The analyses were repeated separately for infants (0 to 5 years), children (6 to 11 years) and adolescents (12 to 14 years) to take into account possible structural variance across age groups.

Models with the best results in the EFA were studied in more depth using confirmatory factor analysis. Pearson correlation coefficients were used to determine the degree of correlation between domains; some degree of correlation between factors was expected.

The descriptive and reliability analysis was conducted based on both the questionnaire structure as administered and the new structure stemming from factor analysis. Score distributions for the 17 items of the PCAT domains was carried out by calculating the mean and standard deviation, the proportion of missing responses, the observed score range, and floor and ceiling effects (i.e. the proportion of cases with the worst and best possible score, respectively). The percentage of items which correlated higher with their hypothesized domain than with the other domains obtained in the EFA (scaling success rates) was calculated. Homogeneity between items in each factor derived from the factor analysis was assessed using Cronbach's alpha.

General satisfaction with services was used to assess construct validity. Satisfaction was analyzed by an item in the main survey questionnaire with four-point Likert-type response scale where 1 = very satisfied, and 4 = very dissatisfied [21]. Validity was assessed by using individual linear regression models with each factor of the PCAT to predict the dissatisfaction score. We expected to find a moderate association between user satisfaction scores and scores derived for each factor of the PC experience scales. Associations were expected to be negative because satisfaction is scored inversely (1 = satisfied) to the PC experience scales (1 = negative).

SPSS 15.0 was used for the descriptive analysis and Mplus version 5.2 was used for the factorial and regression analyses.

## Results

A total of 2.091 parents identified a usual source of care (92.5%), and 4 others (2.7%) had visited a health professional during the previous year. Thus 2.095 parents responded about their experiences with primary care for their children. Sample distribution by age and sex was equivalent to that of the Catalan general population [22]. The proportion of parents of adolescents who were excluded from analysis because they did not identify a usual source of care was higher than in those included in the study, as was the proportion of parents who declared that their child had a chronic condition (table 1). Other differences between these two groups by sex, educational level or type of health care coverage were not statistically significant.

**Table 1:** Demographic and heath characteristics of the sample.

	Identifying an usual source of care					
	
	Yes		No		Total	
**Age**^a^	**n**	**%**	**n**	**%**	**n**	**%**
0 - 5 years	706	33.7	29	27.6	735	33.4
6 - 11 years	927	44.2	39	37.2	966	43.9
12 - 14 years	462	22.1	37	35.2	499	22.7
**Sex**						
Boys	1077	51.4	56	53.3	1133	51.5
Girls	1018	48.6	49	46.7	1067	48.5
**Educational level**. Maximum for father or mother.						
Less than primary	278	13.3	19	18.1	297	13.5
Primary school	530	25.3	24	22.9	554	25.2
Secondary school	643	30.7	42	40.0	685	31.1
University degree	644	30.7	20	19.0	664	30.2
**Health coverage**						
Only public	1591	75.9	82	78.8	1673	76.1
Double (private and public)	504	24.1	22	21.2	526	23.9
**Perceived child's health**						
Good	2032	97.0	104	100.0	2136	97.1
Poor	63	3.0	0	0.0	63	2.9
**Declared chronic conditions**^a^						
Yes	783	37.4	26	25.0	809	36.8
No	1313	62.6	78	75.0	1391	63.2

Comparison between respondents who identified a usual source of care and those who did not.

^a ^p < 0.05

The exploratory factor analysis was conducted to investigate several possible solutions. The goodness of fit of the models was acceptable for all solutions between one and six factors, and the hypothesis of overall scale unidimensionality was rejected (Chi2 = 46.69, gl = 12, p < .001). The model of the six original scales (first contact, ongoing care, coordination, comprehensiveness of services available, comprehensiveness services received, and cultural competence) showed good results, but Heywood cases were observed, and some items loaded onto more than one factor. Heywood cases appear whenever factor loading estimates exceed 1, which implies that unique factors have negative error variances. These cases are likely to occur when there are too many common factors or when there are too many factors to provide stable estimates with the available data. The solution which best fitted the conceptual model was a five factor model with an RMSEA of 0.000, a CFI of 1.00, a TLI of 1.03, and a Chi2 = 3.70 (gl = 6, p = 0.72). In this model, most items loaded highest on the domains they were assigned to in the conceptual model. The exception was the first factor, in which the first contact and ongoing care domains merged (table 2). Another model with acceptable goodness of fit was the most parsimonious, with two factors (RMSEA = 0.023; CFI = 0.95; TLI = 0.92; Chi2 = 21.28, gl = 12, p = 0.019). In this case, the majority of items were grouped within the first factor, whilst a second factor consisted of items measuring services available (comprehensiveness).

**Table 2:** Exploratory factor analysis for the PCAT-CE, very short version.

**Itmes**^**a**^	Domains and items	Extracted factors				
		1	2	3	4	5
	FIRST CONTACT ACCESSIBILITY AND ONGOING CARE					
**B2**	When your child has a new health problem, do you go to your PCP before going somewhere else?	**0.347**	0.088	-0.035	0.017	0.080
**C3**	When your PCP is *open *and your child gets sick, would someone from there see him/her the same day?	**0.474**	-0.195	0.012	0.172	0.098
**C4**	When your PCP is *open*, can you get advice quickly over the phone if you need it?	**0.816**	0.031	0.064	0.091	0.004
**C5**	When your PCP is *closed*, is there a phone number you can call when your child gets sick?	**0.467**	0.154	0.287	-0.186	-0.062
**D1**	When you take your child to your PCP's, is s/he taken care of by the *same *doctor or nurse each time?	**0.309**	0.252	-0.109	-0.140	0.275
**D4**	If you have a question, can you call and talk to *the doctor or nurse who knows your child best*?	**0.741**	0.036	-0.001	0.077	0.131
**D9**	Does your PCP know what problems are most important to you and your family?	**0.388**	0.316	0.070	0.063	0.228
	COORDINATION					
**E10**	Did your PCP write down any information for the specialist about the reason for the visit?	-0.066	**0.869**	0.031	0.026	-0.010
**E12**	After your child went to the specialist or special service, did your PCP talk with you and your child about what happened at the visit?	0.076	**0.866**	-0.070	0.070	0.043
	COMPREHENSIVENESS (SERVICES AVAILABLE)					
**G2**	Immunizations (shots)	-0.417	0.050	**0.532**	0.115	0.144
**G6**	Family planning or birth control methods	0.037	0.023	**0.898**	-0.065	-0.067
**G8**	Counseling for behavior or mental health problems	0.080	-0.087	**0.809**	0.104	0.050
**G10**	Sewing up a cut that needs stitches	0.010	-0.029	**0.574**	-0.038	0.088
	COMPREHENSIVENESS (SERVICES PROVIDED)					
**H1**	Ways to keep your children healthy, such as nutritional foods or getting enough sleep	0.033	-0.010	-0.022	**0.999**	0.016
**H14**	Ways to handle problems with your child's behavior	0.012	0.152	0.067	**0.742**	-0.039
	CULTURALLY COMPETENT					
**K1**	Would you recommend your child's PCP to a friend or relative who has a child?	0.053	0.117	0.016	0.035	**0.824**
**K2**	Would you recommend your child's PCP to someone who does not speak English well?	-0.011	-0.045	-0.002	-0.014	**0.985**

Oblimim rotated factor loadings (highest loadings for each factor in bold).

^a ^Items are presented using the original wording from the US version.

**Figure 1:**
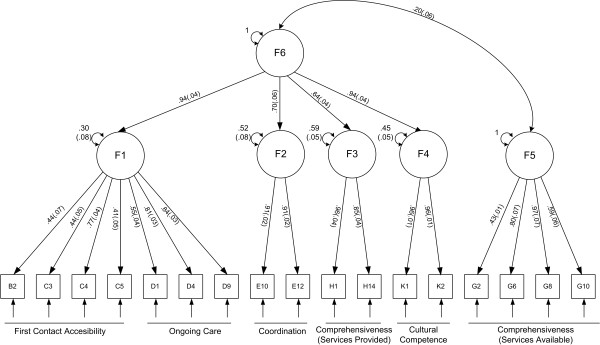
**Factor structure of the PCAT-CE, very short version (confirmatory factor analysis)**. Squares are the items; circles are the resulting factors; unidirectional arrows are factor loadings; and bidirectional arrows are either correlations between factors or residual variances. Standard errors are shown in parenthesis.

Confirmatory factor analysis was conducted for the two models showing the best results in the previous exploratory phase. The five-factor model generated a second order factor which could be defined as "experiences with the content of PC" (F6, see Figure 1) and onto which F1 (first contact accessibility and ongoing care), F2 (coordination), F3 (services received), and F4 (cultural competence) all loaded. Only the services available domain did not load onto this 2nd order factor (F5). This "five plus one" model showed excellent goodness of fit (RMSEA = 0.01, CFI = 0.98; TLI = 0.97; Chi2 = 14.50, gl = 11, p = 0.20) and loadings were high for all six factors. The bifactorial model also presented good results in the CFA (RMSEA = 0.02; CFI = 0.94; TLI = 0.92; Chi2 = 22.86, gl = 11, p = 0.02) with items loading onto factors in a similar fashion to that observed in the EFA and the five plus one model. The multi-group analysis conducted to study the invariance of the structure between age groups confirmed an equivalent factorial structure within the three age groups tested, for the five plus one (Chi2 = 75.0, gl = 53, p = 0.03), and the bifactorial (Chi2 = 67.77, gl = 53, p = 0.06) solutions.

Score distributions by domain and the results of the reliability analysis are shown in table 3. These analyses were carried out for both the six single scales following the structure of the adminstered instrument, and the two new factors obtained from the factorial analysis: the merged first contact accessibility & continuity, and the second order factor ("experience with the content of PC"). Completion rates were high, with no missing responses on any items, except for the coordination domain, which only people who had visited a specialist were required to answer. Means and standard deviations showed generally positive experiences with primary care and distributions were non-normal, with a marked ceiling effect in Coordination and Cultural Competence. Internal consistency was poor (alpha < 0.50) for the First Contact-Accessibility domain and moderate (alpha < 0. 57-0.70) in three of the original six (ongoing care, services available, and services provided). The merged scales resulting from factor analysis showed a lower ceiling effect and achieved satisfactory homogeneity (alpha > 0.70). Scaling success was not achieved in the ongoing care domain, but was satisfactory in the remaining domains (Table 3).

**Table 3:** Score distribution and reliability of the PCAT domains - original and modified scaling.

PCAT domains	Num. of items	**% Com-pletion**^**a**^	Mean (SD)	Observed range	Floor	Ceiling	% Scaling succes	Cronbach's alpha (SE)
First Contact Accessibility	4	100.0	3.40 (0.52)	1.00-4.00	0.0	24.6	100.0	0.439 (0.020)
Ongoing Care	3	100.0	3.25 (0.73)	1.00-4.00	2.1	29.2	33.3	0.572 (0.018)
Coordination	2	57.8	3.43 (0.86)	1.00-4.00	5.5	59.4	100.0	0.702 (0.015)
Comprehensiveness (Services Available)	4	100.0	3.21 (0.51)	1.00-4.00	0.1	12.1	100.0	0.599 (0.016)
Comprehensiveness (Services Provided)	2	100.0	3.03 (0.96)	1.00-4.00	9.9	33.9	100.0	0.666 (0.014)
Cultural Competence	2	100.0	3.50 (0.77)	1.00-4.00	3.8	60.1	100.0	0.832 (0.003)

First Contact Accessibility & Ongoing Care	7	100.0	3.33 (0.52)	1.00-4.00	0.0	14.4	100.0	0.710 (0.012)

Experience with PC (second order factor) ^b^	13	57.8	3.35 (0.55)	1.25-4.00	0.0	5.9	100.0	0.799 (0.011)

^a ^Completion rate: percentage of items responded within the scale from the 2,095 cases responding to the health interview survey.

^b ^The second order factor (F6) is a scale computed from the domains of first contact & ongoing care, coordination, services provided, and cultural competence. It excludes the 'services available' domain.

The PCAT scales showed the expected pattern of association with the overall satisfaction with health-care item, i.e. parents reporting dissatisfaction with healthcare services scored low in most of the PC experiences domains (table 4).

**Table 4:** Construct validity.

PCAT domains	Standardized beta	S.E.	z	p-value
First Contact Accessibility	-0.38	0.03	-11.26	<0.01
Ongoing Care	-0.36	0.03	-11.80	<0.01
Coordination	-0.35	0.04	-9.27	<0.01
Comprehensiveness (Services Available)	-0.49	0.17	-2.92	<0.01
Comprehensiveness (Services Provided)	-0.42	0.05	-8.61	<0.01
Cultural Competence	-0.42	0.03	-13.09	<0.01

First Contact & Ongoing Care	-0.36	0.06	-11.6	<0.01

Experience with PC (second order factor)	-0.35	0.03	-11.06	<0.01

Individual regression models between PCAT domains and general satisfaction with health services.

The second order factor (F6) is a scale computed from the domains of first contact & ongoing care, coordination, services provided, and cultural competence. It excludes the 'services available' domain.

## Discussion

This study analyzed the structure, construct validity and reliability of a very short version of the PCAT for parents of children aged 0 - 14 years. This instrument was derived from the original short version developed by researchers in the US (PCAT-CS), and its seventeen items measure several attributes of PC, based on the questionnaire's original conceptual model, with acceptable reliability and validity.

An important finding from the study included the fact that items about ongoing care correlated highly with those measuring first contact accessibility, to the extent that they all loaded onto a single factor. The original PCAT 'ongoing care' domain contained two types of items: those concerning identification with a particular provider and those concerning the nature of the relationship. In the very abridged version used in this study, 2 of the 3 items from the subdomain of interpersonal relationships were associated, making them similar in concept to the items on access in the first-contact subdomain. There is thus a difference with the original instrument, since the smaller number of items and the traits captured by them cannot collect equal information addressing the nature of the relationship, i.e., person focused care over time. Apart from this limitation, the analysis of structural validity indicated that the items selected adequately represent the original conceptual multidimensional model because data fitted well to the remaining 4 domains of coordination, services available, services provided, and cultural competence.

The bifactorial model was also confirmed as an alternative measure of two concepts, with good statistical fit. 'Services available' (a list of characteristics of the source of care that might be needed at some time) is different from services received and, hence, not strictly an 'experiences' measure. Moreover, the second order factor resulting from the confirmatory factor and its reliability analysis underlined the possibility of gathering the information on a unique scale. This option might be useful when a single global measure is required, and could improve the metric properties of the four original scales. Nevertheless, content validity in terms of the theoretically expected attributes of PC would be reduced as this scale retains fewer than half of the original items. Moreover, if this overall scale is computed it would not include the items relating to the comprehensiveness of primary care services available and would not apply to individuals who did not visit a specialist during the previous year, as they are not required to respond to the questions on coordination.

Using the original scale structure as the 'gold standard', reliability as measured using Cronbach's alpha was suboptimal for some of the individual scales. However, these results derive from a very small subset of the original scales, which makes it difficult to achieve a desired level of internal consistency reliability. When we calculated reliability based on the new factor structure, the alpha coefficients improved considerably suggesting that it would be more appropriate to base scoring of this reduced version on the factorial structure observed in the present study.

The construct validity analysis confirmed the expected pattern of association between experiences with PC and the item assessing satisfaction with health services. Due to a lack of instruments in Spanish measuring similar concepts, we were unable to assess convergent validity. This is a point which should be addressed in the future if such instruments become available. Another aspect which was not assessed was test-retest reliability, which would provide evidence of the degree of random error between two measures from the same individual.

The items used in this very short version of the PCAT were adapted for use in Spain following internationally recommended principles and methods for cross-cultural adaptation to maintain semantic equivalence between language versions. This makes it possible to compare results obtained here with those from the US or other countries in which the same subset of PCAT items has been applied.

## Conclusions

The PCAT set of instruments allows for a variety of possibilities in health services research aimed at informing policy makers about the adequacy of different attributes of primary care. The Catalan Health Survey was the first large scale study including a selection of items from PCAT to assess the quality of Primary Health Care. This selection of items for a population health survey is particularly important because it provides a population perspective and can be useful to study inequalities in experiences with fundamental attributes of primary care. This very short questionnaire could be useful as a measure of primary care experiences if space is at a premium, as is often the case in population health surveys. Based on preliminary evidence of construct validity and reliability provides a summary score of judgments about experiences with primary care overall. It also can report scores on five domains of the theoretical multidimensional model on the base of their content validity and acceptable metric properties, even though lower reliability in some domains was observed and must be took into account. However, the loss of content stemming from the use of a subset of items, argues for the use of the longer versions of the PCAT to capture all the expected domains of primary care, when possible.

## Competing interests

The authors declare that they have no competing interests.

## Authors' contributions

SB and MIP drew up the initial project design; MRS, LR, CB, BS participated in the study design and contributed to the data analysis plan; KBR carried out part of the statistical analysis and the first draft of the results; SB drafted the manuscript; all authors revised it critically, made contributions to the interpretation of data, and gave final approval of the version to be published.

## Pre-publication history

The pre-publication history for this paper can be accessed here:

http://www.biomedcentral.com/1471-2458/11/285/prepub
